# Phylogenetic relationships of sucrose transporters (SUTs) in plants and genome-wide characterization of *SUT* genes in Orchidaceae reveal roles in floral organ development

**DOI:** 10.7717/peerj.11961

**Published:** 2021-09-13

**Authors:** Yunzhu Wang, Yue Chen, Qingzhen Wei, Hongjian Wan, Chongbo Sun

**Affiliations:** 1 Institute of Horticulture Research, Zhejiang Academy of Agricultural Sciences, Hangzhou, China; 2 Institute of Vegetable Research, Zhejiang Academy of Agricultural Sciences, Hangzhou, China

**Keywords:** Sucrose transporters, Orchidaceae, Gene family, Water-soluble sugar content, Gene expression

## Abstract

Sucrose is the primary form of photosynthetically produced carbohydrates transported long distance in many plant species and substantially affects plant growth, development and physiology. Sucrose transporters (SUTs or SUCs) are a group of membrane proteins that play vital roles in mediating sucrose allocation within cells and at the whole-plant level. In this study, we investigated the relationships among SUTs in 24 representative plant species and performed an analysis of *SUT* genes in three sequenced Orchidaceae species: *Dendrobium officinale*, *Phalaenopsis equestris*, and *Apostasia shenzhenica*. All the SUTs from the 24 plant species were classified into three groups and five subgroups, subgroups A, B1, B2.1, B2.2, and C, based on their evolutionary relationships. A total of 22 *SUT* genes were identified among Orchidaceae species, among which *D. officinale* had 8 genes (*DoSUT01-08*), *P. equestris* had eight genes (*PeqSUT01-08*) and *A. shenzhenica* had 6 genes (*AsSUT01-06*). For the 22 Orchidaceae*SUTs*, subgroups A, B2.2 and C contained three genes, whereas the *SUT* genes were found to have significantly expanded in the monocot-specific subgroup B2.1, which contained 12 genes. To understand sucrose partitioning and the functions of sucrose transporters in Orchidaceae species, we analyzed the water-soluble sugar content and performed RNA sequencing of different tissues of *D. officinale*, including leaves, stems, flowers and roots. The results showed that although the total content of water-soluble polysaccharides was highest in the stems of *D. officinale*, the sucrose content was highest in the flowers. Moreover, gene expression analysis showed that most of the *DoSUTs* were expressed in the flowers, among which *DoSUT01*,*DoSUT07* and *DoSUT06* had significantly increased expression levels. These results indicated that stems are used as the main storage sinks for photosynthetically produced sugar in *D. officinale* and that *DoSUTs* mainly function in the cellular machinery and development of floral organs. Our findings provide valuable information on sucrose partitioning and the evolution and functions of *SUT* genes in Orchidaceae and other species.

## Introduction

Photoassimilated carbohydrates are produced by autotrophic source tissues such as leaves and are translocated to heterotrophic sink tissues such as roots, stems, flowers and seeds. Sucrose is the major transported form of photosynthetically produced sugar in many plant species due to its nonreducing nature and insensitivity to degradation (Lemoine, 2000). Long-distance sucrose transport in the phloem requires transmembrane transport. Sucrose transporters (SUTs or SUCs) play vital roles in transmembrane transport during phloem loading and unloading as well as in sucrose allocation within plants and between pathogens and beneficial symbionts (Kühn & Grof, 2010).

Plant sucrose transporters are members of the major facilitator superfamily (MFS), which typically have 12 transmembrane (TM) domains divided into two regions by a hydrophilic cytoplasmic loop (Lalonde, Wipf & Frommer, 2004; Bush, 1993). All of the transporters in the SUT/SUC family studied to date are sucrose/H+ symporters, including vacuolar SUTs. The first plant *SUT* gene, *SoSUT1*, was isolated from spinach using a yeast complementation system (Riesmeier, Willmitzer & Frommer, 1992). With the increasing availability of plant genomes and molecular information, a growing number of *SUT* s have been identified in many plant species, including both monocot and dicot species, such as *Arabidopsis* (Weise et al., 2000), rice (Aoki et al., 2003), *Populus* (Hackel et al., 2006; Payyavula et al., 2011), wheat (Deol et al., 2013), maize (Usha, 2015), pear (Zhang et al., 2013), cacao (Li et al., 2014), tomato (Reuscher et al., 2014), cotton (Li et al., 2018) and various species considered weeds (Misra et al., 2019). However, these genes are absent in the unicellular chlorophyte alga *Chlamydomonas reinhardtii* and in *Volvox carteri* (Reinders, Sivitz & Ward, 2012).

According to the genomes of grasses, *SUT* genes were originally classified into five groups: SUT1-SUT5 (Kühn & Grof, 2010; Braun & Barker, 2009; Lalonde & Frommer, 2012). The SUT1 clade is dicot specific, with members expressed in the plasma membrane of sieve elements or companion cells (Weise et al., 2000; Stadler et al., 1995; Baker et al., 2016). SUT2 and SUT4 encompass members from both dicot and monocot plants, whereas both SUT3 and SUT5 groups are monocot specific. SUT2 transporters are mainly expressed in the plasma membrane of SEs and are found in vegetative sink organs (Barth, Meyer & Sauer, 2003; Meyer et al., 2004). All members of the SUT4 clade are proposed to be vacuolar SUTs (Endler et al., 2006; Chincinska et al., 2008). Recently, researchers have divided *SUT* s into two subfamilies (Ancient Group 1 and Ancient Group 2) and three types (type I, type II and type III) (Reinders, Sivitz & Ward, 2012; Peng et al., 2014). *SUT* family genes play essential roles in phloem loading and unloading, pollen development, fruit ripening, ethylene biosynthesis and seed development and germination in many plant species (Payyavula et al., 2011; Usha, 2015; Chincinska et al., 2008; Sivitz, Reinders & Ward, 2008; Srivastava et al., 2009a). In addition, *SUT* genes are involved in various physiological processes and sucrose exchange between plants and symbionts, pathogens and fungi (Kühn & Grof, 2010; Doidy et al., 2012; Wittek et al., 2017). For example, in *Arabidopsis*, AtSUC5 is predominantly expressed in the seeds. AtSUC1 is expressed in seedlings, where it is necessary for normal anthocyanin accumulation. AtSUC1 is also expressed in pollen and required for normal pollen function. AtSUC9 appears to be required for normal floral transition (Sivitz et al., 2007; Sivitz, Reinders & Ward, 2008), and *OsSUT2* is expressed in the seeds and is involved in the germination of embryos (Siao et al., 2011; Eom et al., 2016). The activity and expression of sucrose transporters are regulated by genetic, molecular and physiological factors.

Orchidaceae is one of the largest families in angiosperms, with more than 25,000 species and 880 genera, representing ∼10% of flowering plants (Sharma & Mukai, 2015). Many of them are economically important due to their unique ornamental and medicinal value. Moreover, orchids are model systems for elucidating floral evolution in angiosperms and symbiotic activities between plants and fungi (Hsiao et al., 2011; Zhang et al., 2017). To date, the genomes of three Orchidaceae species, *Dendrobium officinale*, *Phalaenopsis equestris*, and *Apostasia shenzhenica*, have been sequenced and published, which has greatly promoted the understanding of the genetics and genomics of orchids (Zhang et al., 2016; Zhang et al., 2017; Yan et al., 2015). However, the roles of sucrose transporters in orchids are still unknown. In the present study, we performed genome-wide identification and characterization of the members of the *SUT* gene families in three sequenced Orchidaceae species. Transcriptome sequencing and water-soluble sugar content analysis were also conducted in *D. officinale*. Our findings provide insight into the evolution, expression, and functions of *SUT* genes in Orchidaceae.

## Material and Methods

### Identification and characterization of SUT proteins in Orchidaceae

The genome, gene and corresponding protein sequences of three sequenced Orchidaceae species, *D. officinale* (Zhang et al., 2016; Yan et al., 2015), *P. equestris* (Cai et al., 2014), and *A. shenzhenica* (Zhang et al., 2017), were downloaded from NCBI (https://www.ncbi.nlm.nih.gov/assembly/GCF_001605985.2/) and OrchidBase (http://orchidbase.itps.ncku.edu.tw/est/home2012.aspx). All members of the SUT family contain the GPH_sucrose (TIGR01301) domain, the seed sequence of which was downloaded from the TIGRFAMS database (http://tigrfams.jcvi.org/cgi-bin/index.cgi). ClustalW (Thompson, Gibson & Higgins, 2003) was used for sequence alignment, and a hidden Markov model (HMM) (Eddy, 1998) was constructed for SUT proteins. The HMMER program was used to search for SUT proteins among all *D. officinale*, *P. equestris*, and *A. shenzhenica* proteins, with a cutoff *E*-value of 1e ^−4^, using the HMM as a query. If the location of two *SUT* genes in the genome was less than 10 kb part, they were considered homologous genes generated by fragment duplication; if not, they were considered homologous genes generated by genome-wide duplication. After a comprehensive check, the candidate proteins that contained only fragmented SUT domains were eliminated. The ProtParam (http://web.expasy.org/protparam/) website was used to determine the molecular weight of each gene, and the theoretical isoelectric point (pI) of each protein was also predicted.

### Phylogenetic analysis of SUT proteins

The amino acid sequences of SUT proteins identified in three Orchidaceae species (*A. shenzhenica*, *D. officinale*, *P. equestris*) and 21 other species were used in a phylogenetic analysis that included algae, moss, lycophytes, and angiosperms: *Chlamydomonas reinhardtii* (*Cre*), *Volvox carteri* (*Vca*), *Physcomitrella patens* (*Ppa*), *Selaginella moellendorffii* (*Smo*), *Aquilegia coerulea* (*Aco*), *Picea abies* (*Pab*), *Brachypodium distachyon* (*Bdi*), *Oryza sativa* (*Osa*), *Zea mays* (*Zma*), *Vitis vinifera* (*Vvi*), *Eucalyptus grandis* (*Egr*), *Malus domestica* (*Mdo*), *Carica papaya* (*Cpa*), *Cucumis sativa* (*Csa*), *Daucus carota* (*Dca*), *Solanum lycopersicum* (*Sly*), *Asparagus officinalis* (*Aof*), *Populus trichocarpa* (*Ptr*), *Arabidopsis thaliana* (*AT*), *Glycine max* (*Gma*), and *Theobroma cacao* (*Tca*). The protein sequences were downloaded from the Pfam database (https://phytozome.jgi.doe.gov/) and Phytozome database (https://phytozome.jgi. doe.gov/). MEGA 6 (V6.0, Tokyo Metropolitan University, Tokyo, Japan) was used to systematically analyze the protein sequences of the SUTs. First, CLUSX2 in MEGA 6 was used for multiple sequence alignment, and then the maximum likelihood (ML) method with the Jones-Taylor-Thornton (JTT) model was used to construct a phylogenetic tree. Moreover, 1,000 bootstrap replicates and a partial deletion with a site coverage cutoff of 70% were used for gap treatment. The phylogenetic trees were visualized using FigTree v1.4.2 (http://tree.bio.ed. ac.uk/software/figtree/).

### Gene structure and motif analyses

The Gene Structure Display Server tool (http://gsds.cbi.pku.edu.cn/; v2.0) was used to analyze the gene structure of all the SUTs identified in *D. officinale*, *P. equestris*, and *A. shenzhenica*. MEME software (http://meme.nbcr.net/meme; v4.11.0) was then used to search for motifs in SUT proteins, with motif window lengths from 10 to 100 bp; the maximum number of motifs was set at 20, and motifs present in at least three SUT proteins were identified as true motifs.

### Analysis of SUT gene expression in different tissues of *D. officinale*

First, we performed RNA-seq on different tissues of *D. officinale*. Three-year-old *D. officinale* plants were grown in glasshouses at the Mulberry Field Station of Zhejiang Academy of Agriculture Science (Hangzhou, China). Four different tissues—roots, stems, leaves and flowers—were collected, frozen in liquid nitrogen, and then stored at −80 °C until use. Each tissue was sampled three independent times. Total RNA was extracted using TRIzol reagent (Invitrogen, Carlsbad, CA, USA) according to the manufacturer’s instructions. The library preparations were sequenced on an Illumina HiSeq 2000 platform (Illumina, Inc; San Diego, CA, U.S.), and 150 bp paired-end reads were generated for the 12 samples. Then, the expression profiles of all the *Dendrobium* genes were obtained *via* fragments per kilobase of exon per million fragments mapped (FPKM) values using Cufflinks software (http://cole-trapnell-lab.github.io/cufflink; v2.2.1) under the guidance of annotated gene models with a GFF file. The *SUT* gene expression profile from each sample was analyzed using the HemI program (http://hemi.biocuckoo.org/) with the average hierarchical clustering method.

### Determination of the total water-soluble polysaccharide content

Three-year-old *D. officinale* plants were grown in glasshouses at the Mulberry Field Station of Zhejiang Academy of Agriculture Science (Hangzhou, China). Four *D. officinale* tissues—roots, stems, leaves and flowers (three replicates for each tissue)—were collected and dried in an oven at 105 °C until a constant weight was achieved. The 12 samples were independently ground into fine powders by a mixing mill (MM 400, Retsch). Total polysaccharides were extracted using the water extraction and alcohol precipitation methods, and the content of total polysaccharides was measured using the phenol-sulfuric acid method.

Total polysaccharide extraction: Approximately 0.05 g of each sample was weighed, added to one mL of water, and fully homogenized. Each sample was then extracted in a water bath at 100 °C for 2 h and subsequently centrifuged at 10000 × g for 10 min after cooling, and the supernatant was removed. Then, 0.2 mL of the supernatant was collected, and 0.8 mL of anhydrous ethanol was slowly added. After mixing, the mixture was stored overnight at 4 °C. After centrifugation at 10,000 g for 10 min, the supernatant was discarded, and one mL of water was added to the precipitate, after which the mixture was thoroughly mixed and dissolved.

To calculate the total polysaccharide content, the microplate reader was preheated for more than 30 min, and the wavelength was adjusted to 490 nm. Two hundred microliters of the supernatant was extracted, and 100 µL of the reagent and 0.5 mL of concentrated sulfuric acid were added. After the contents of the wells were mixed together, the mixtures were incubated in a 90 °C water for 20 min. A 200 µL mixture was extracted and added to an enzyme-labeled plate, and the absorbance value (A) was determined at 490 nm. Glucose was used as a reference. The regression equation under standard conditions was *y* = 7.981x−0.0037, R2 = 0.9973; were, x represents the glucose content (mg/mL), and y represents the absorbance value. The total polysaccharide content (µg/g dry weight) was calculated as (A+0.0037) ÷7. 981 ×V1 ÷V 2 ×V3 ÷W ×1000 = 626.49 ×(A + 0.0037) ÷W. Here, V1 is the redissolved volume after alcohol precipitation (one mL); V2 is the volume of alcohol precipitation (0.2 mL); V3 is the volume of water added during extraction (one mL); W is the sample weight (g); and 1000 is the conversion coefficient for milligrams to micrograms.

### Determination of the sucrose content

After drying, the 12 samples were ground into a fine powder independently with a mixing mill (MM 400; Retsch). Twenty milligrams of powder was diluted in 500 µL of a methanol:isopropanol:water (3:3:2 v/v/v) solution. The extract was centrifuged at 14,000 rpm at 4 °C for 3 min. Fifty microliters of the supernatant and an internal standard (Shanghai ZZBIO Co., Ltd.) were subsequently mixed together, evaporated under a stream of nitrogen gas, and then transferred to a lyophilizer for freeze drying. The residue was subjected to further derivatization. A sample of small-molecule carbohydrates and a 100 µL solution of methoxyamine hydrochloride in pyridine (15 mg/mL) were then mixed together. The mixture was incubated at 37 °C for 2 h. Then, 100 µL of BSTFA was added to the mixture and incubated at 37 ° C for 30 min after vortexing. The mixture was subsequently diluted and analyzed *via* GC-MS/MS according to the methods of Gómez-González et al. (2010) and Sun et al. (2016), with modifications. An Agilent 7890B gas chromatograph coupled to a 7000D mass spectrometer equipped with a DB-5 MS column (30 m length ×0.25 mm i.d. ×0.25 µm film thickness, J&W Scientific, USA) was used for GC-MS/MS analysis of the sugars. Helium was used as the carrier gas at a flow rate of one mL/min. The injections were made in split mode at a ratio of 3:1, and the injection volume was 3 µL. The oven temperature was set at 170 °C for 2 min, raised to 240 °C at 10 ° C/min, raised to 280 °C at 5 °C/min, raised to 310 °C at 25 °C/min and then held for 4 min. All the samples were analyzed in selective ion monitoring mode. The injector inlet and transfer line temperatures were 250 °C and 240 °C, respectively.

### RNA extraction and qRT-PCR analyses

Total RNA was extracted from three *D. officinale* tissues, flowers, stems and leaves, using TRIzol reagent (Invitrogen, Carlsbad, CA, USA) according to the manufacturer’s instructions. DNase I was used to purify potential contaminated genomic DNA. The quality of total RNA was checked with 1% denaturing agarose gels and a NanoDrop 2000 spectrophotometer (Thermo Fisher Scientific, Beijing, China). First-strand cDNA synthesis was performed with PrimeScript reverse transcriptase (TaKaRa Biotechnology, Dalian, China), with RNA used as the template. Gene-specific primers were designed with the Primer Premier 5.0 program (Table S3). The *DnActin* (comp205612_c0) gene was used as an internal standard for normalizing the gene expression data (Chen et al., 2017). The expression levels of *DoSUTs* were analyzed *via* a qRT-PCR assay, which was completed with a SYBR Green qPCR kit (TaKaRa Biotechnology, Dalian, China) and a Stratagene Mx3000P thermocycler (Agilent, Santa Clara, CA, USA). The PCR program was as follows: 95 °C for 10 min, followed by 40 cycles of 95 °C for 15 s and 60 °C for 60 s. The relative *SUT* gene expression levels were calculated with the 2^−ΔΔCt^ method (Livak & Schmittgen, 2001). The analysis included three biological replicates, each with three technical replicates (Table S4). The expression levels in the different tissues were visualized with a histogram using the average values.

### Statistical analysis

Statistical analysis was performed to calculate the average values and standard errors of three replicates. SPSS software (*v.* 16.0) was used to determine the significant differences in sugar content among the different tissues using one-way ANOVA and post hoc analysis. *P* value = 0.05 indicates a significant difference and is represented by an asterisk (*) in the figures; *p* value = 0.01 indicates a very significant difference and is represented by two asterisks (**).

## Results

### Genome-wide identification of SUT genes in Orchidaceae species

To understand the potential roles of SUTs in orchids, three sequenced Orchidaceae species, *D. officinale*, *P. equestris*, and *A. shenzhenica*, were used for genome-wide identification and characterization of *SUT* genes. The HHM profile of the SUT proteins was used as a query to perform an HMMER search against the genome assemblies of the three species. Bioinformatics analysis identified a total of 22 *SUT* s with different serial numbers from the three species, which were designated ‘*DoSUT*’ for *D. officinale*, ‘*PeqSUT*’ for *P. equestris*, and ‘*AsSUT*’ for *A. shenzhenica* (Table 1, Table S1). Among them, *D. officinale* had eight genes (*DoSUT01-08*), *P. equestris* had eight genes (*PeqSUT01-08*) and *A. shenzhenica* had six genes (*AsSUT01-06*). These results agree with those of previous reports that plant sucrose transporters are encoded by relatively small gene families.

**Figure 1:**
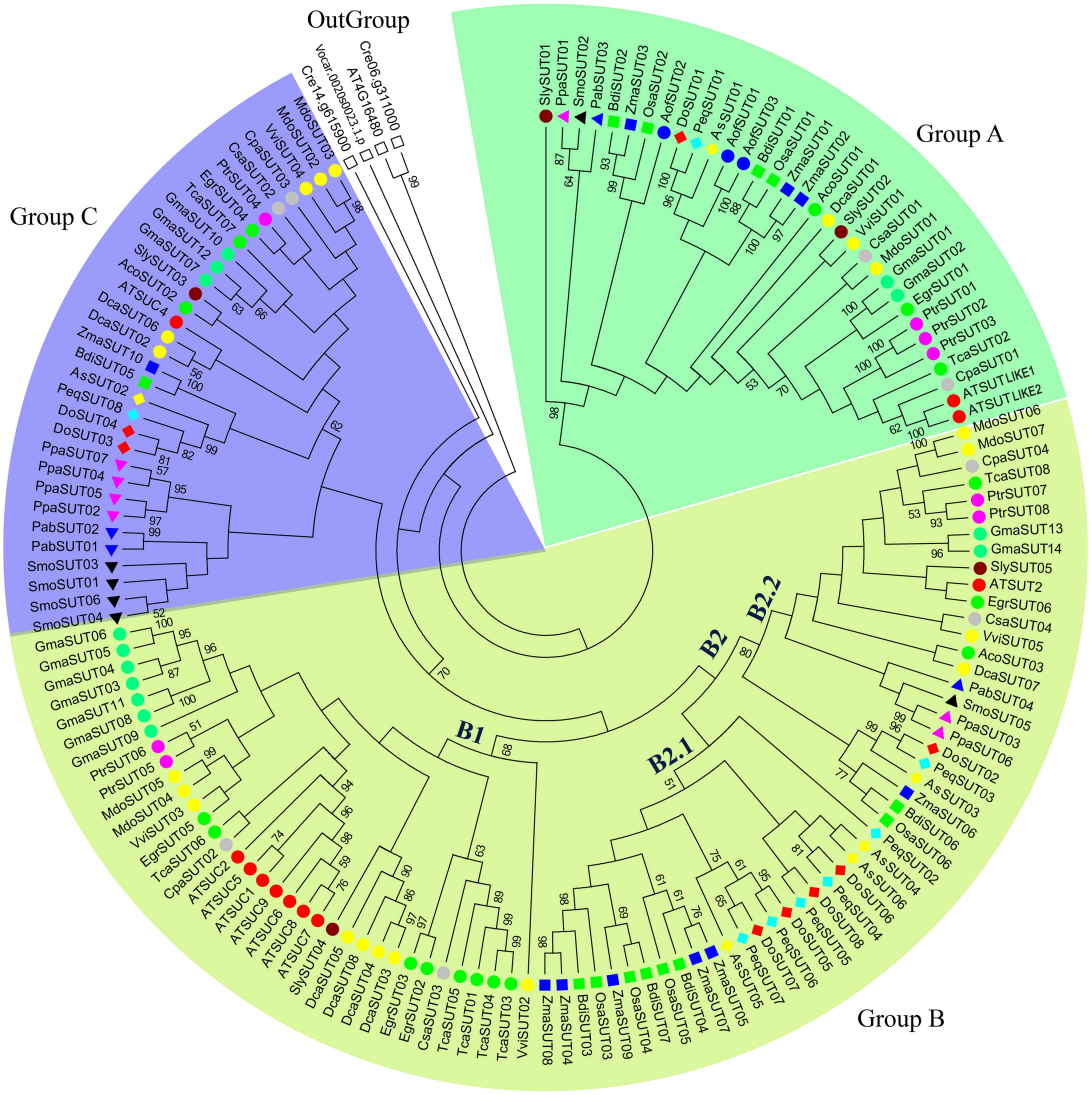
Phylogenetic analysis of *SUT* gene families from 24 representative plant species. The phylogenetic tree was constructed using MEGA 6.0 with the maximum likelihood (ML) method and 1,000 bootstrap replicates. All SUT sequences were grouped into three groups and five subgroups (A, B1, B2.1, B2.2, and C). Green, blue, and yellow green shades indicate groups A, B, and C, respectively. The gene code prefixes are as follows: *Chlamydomonas reinhardtii* (*Cre*), *Volvox carteri* (*Vca*), *Physcomitrella patens* (*Ppa*), *Selaginella moellendorffii* (*Smo*), *Aquilegia coerulea* (*Aco*), *Picea abies* (*Pab*), *A. shenzhennica* (*Apo*), *D. officinale* (*Den*), *P. equestris* (*Peq*), *Brachypodium distachyon* (*Bdi*), *Oryza sativa* (*Osa*), *Zea mays* (*Zma*), *Vitis vinifera* (*Vvi*), *Eucalyptus grandis* (*Egr*), *Malus domestica* (*Mdo*), *Carica papaya* (*Cpa*), *Cucumis sativa* (*Csa*), *Daucus carota* (*Dca*), *Solanum lycopersicum* (*Sly*), *Asparagus officinalis* (*Aof*), *Populus trichocarpa* (*Ptr*), *Arabiqopsis thaliana* (*AT*), *Glycine max* (*Gma*), and *Theobroma cacao* (*Tca*)*.* Individual species are distinguished by circle, triangle, square, or rhombus in different colors.

According to the phylogenetic tree, the 22 *SUT* genes from the three orchids could be classified into four subgroups: subgroups A, B2.1, B2.2 and C (Fig. 1, Table 1). Subgroup A included three genes: *DoSUT01*, *PeqSUT01* and *AsSUT01*. There were four genes in subgroup C (*DoSUT03*, *DoSUT04*, *PeqSUT08* and *AsSUT02*) and three genes in subgroup B2.2 (*DoSUT02*, *PeqSUT03* and *AsSUT03*). However, the *SUT* genes had significantly expanded in the monocot-specific subgroup B2.1, which comprised 12 genes. Phylogenetically, the sucrose transporters in *D. officinale* were more closely related to those in *P. equestris* than to those in *A. shenzhenica*.

The molecular weights of the SUTs ranged from 18.81 to 106.90 kDa, with pI values ranging from 4.95 to 10.12. Most of these genes were ∼500 aa or ∼600 aa in length, with 11–13 introns and 12–14 exons, whereas there were several genes with only 4–5 introns/exons. These findings are consistent with the findings of the present study. Detailed information on the *SUT* genes, including their name, encoded protein, CDS length, molecular weight and PI value, is shown in Table 1.

**Table 1:** Physical and molecular characteristics of *SUT* genes in *A. shenzhenica*, *D. officinale*, and *P. equestris*.

**Gene name**	**Scaffold location** **(bp)**		**Subgroup**	**Length** **(bp)**	**Size** **(aa)**	**MW** **(kDa)**	**pI**	**Exon**	**Intron**
*AsSUT01*	1215667	1232090	A	16423	458	49879.49	7.5	12	11
*AsSUT02*	644564	658003	C	13439	532	56556.26	9.6	5	4
*AsSUT03*	1021836	1041725	B2.2	19889	589	64293.55	5.39	13	12
*AsSUT04*	328657	335790	B2.1	7133	488	51878.16	8.85	13	12
*AsSUT05*	534128	538346	B2.1	4218	477	51224.4	9.05	14	13
*AsSUT06*	314008	320943	B2.1	6935	499	52812.97	8.36	14	13
*DoSUT01*	1939695	1953824	A	14147	716	78785.55	8.55	13	12
*DoSUT02*	11559848	11579293	B2.2	19445	571	62989.08	4.95	14	13
*DoSUT03*	83114	90826	C	7712	216	22730.49	10.12	5	4
*DoSUT04*	74903	90826	C	7712	216	22730.49	10.12	5	4
*DoSUT05*	10800848	10810268	B2.1	9420	984	106898.82	8.93	14	13
*DoSUT06*	3505256	3512511	B2.1	7255	177	18812.02	8.79	4	3
*DoSUT07*	29089	32887	B2.1	3798	492	52983.52	9.06	14	13
*DoSUT08*	397815	406603	B2.1	8788	470	50102.76	8.38	14	13
*PeqSUT01*	247532	268499	A	20967	461	50349.07	7.51	13	12
*PeqSUT02*	14129	22513	B2.1	8384	240	26208.15	9.3	8	7
*PeqSUT03*	3166955	3194083	B2.2	27128	611	65924.14	6.19	14	13
*PeqSUT04*	2062452	2066080	B2.1	3628	499	53546.81	8.32	14	13
*PeqSUT05*	58510	76466	B2.1	17956	500	53040.17	8.24	14	13
*PeqSUT06*	523303	531370	B2.1	8067	492	52933.39	9.11	14	13
*PeqSUT07*	659976	662642	B2.1	2666	489	52440.8	9.17	12	11
*PeqSUT08*	10082423	10107132	C	24709	413	43952.87	9.02	7	6

### Phylogenetic relationships of SUT proteins in major plant species

In the present study, the evolution of SUT gene families among the representative plant species was systematically investigated. A phylogenetic tree comprising 24 plant species was constructed, including green algae, mosses, lycophytes, gymnosperms, monocots and dicots. The SUT domain sequence and neighbor-joining method were used to construct the phylogenetic tree, with 1,000 bootstrap replicates. In this study, the SUT genes of several eukaryotic chlorophytes clustered on a unique branch, which was defined as an outgroup. All SUTs were classified into three groups and five subgroups—A, B1, B2.1, B2.2, and C (Fig. 1). Group A contained at least one member from mosses, lycophytes and angiosperms, including both monocots and dicots. Group B was the largest group and was divided into three subgroups; subgroup B1 comprised SUTs exclusively from dicot species, corresponding to the SUT1 clade (Lalonde & Frommer, 2012). Subgroup B2.2 contained SUTs from both monocot and dicot species that were also present in the SUT2 group (Lalonde & Frommer, 2012). Subgroup B2.1 was a monocot-specific expansion clade containing SUT3 and SUT5, as reported by Kühn & Grof (2010). Group C contained SUTs from mosses, lycophytes and angiosperms, including both monocots and dicots, corresponding to the SUT4 clade (Lalonde & Frommer, 2012). Type I SUTs have typically been proposed to be specific to eudicots. Notably, no orchid SUTs were found in clade B1; however, they were found in both clades B2.1 and B2.2.

Sucrose transporters have been identified in lower terrestrial plants, including both lycophytes and mosses, with six *SUTs* in *Selaginella lepidophylla* and 7 *SUTs* in *Physcomitrella patens*. There were 6-10 *SUT* genes in monocot species such as rice (six genes), maize (10 genes) and sorghum (eight genes). In contrast, in another monocot species, *Ananas comosus*, only three *SUTs* were identified. For most dicot species, 4-9 *SUTs* were identified. These results revealed that the number of sucrose transporters remained largely stable during the evolution from lower plants to terrestrial plants. However, the *SUTs* expanded in several species, such as *Triticum aestivum* (18 genes) and *Glycine max* (14 genes), which may be the result of whole-genome polyploidization. The *SUT* s of some monocot species expanded in subgroup B2.1; for example, there were five *ZmaSUT* s in subgroup B2.1, whereas 3 *ZmaSUT* s were identified in subgroup A, and only one was identified in subgroups B2.2 and C. Likewise, the *SUTs* from dicot species, such as *GmaSUT* s, *AtSUT* s and *DcaSUT* s, expanded in subgroup B1. The characean alga *Chlorokybus atmosphyticus* contains one *SUT* homolog that is basal to all streptophyte *SUTs* (Reinders, Sivitz & Ward, 2012). We also identified one *SUT* (*VcaSUT01*) in the chlorophyte *Volvox carteri*. Therefore, the origin of sucrose transporters predates the divergence between green algae and the ancestors of terrestrial plants.

### Conserved motif analyses of SUT genes

**Figure 2:**
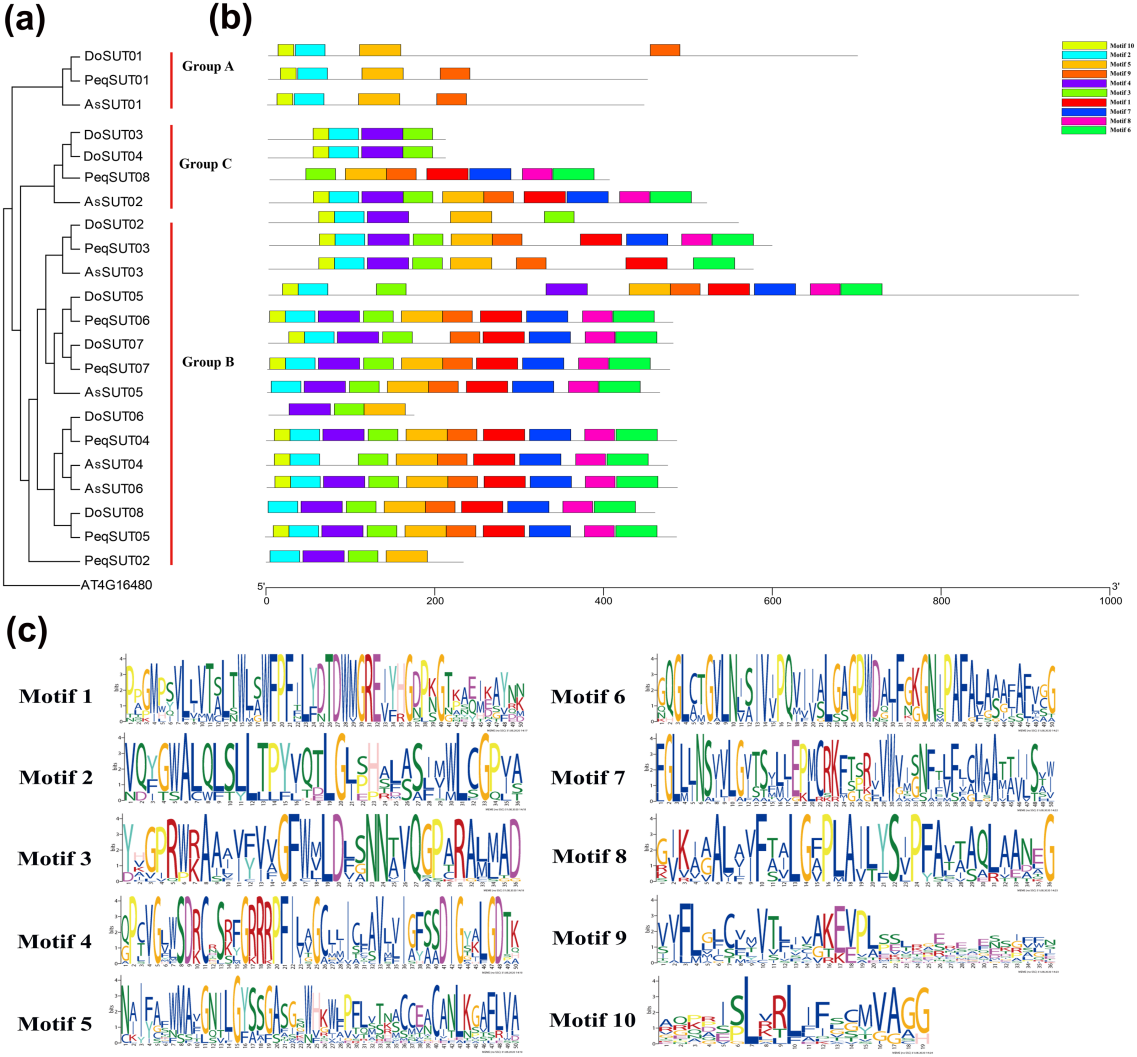
Phylogenetic and conserved motif analyses of the SUT proteins from *A. shenzhennica* (*Apo*), *D. officinale* (*Den*), and *P. equestris* (*Peq*). (A) Phylogenetic tree of the SUT proteins using AT4G16480 as outgroup; (B) schematic presentation of the conserved motifs in Orchidaceae SUTs; (C) sequence logos of all the 10 motifs.

The diversity of motif compositions among sucrose transporters of Orchidaceae species was assessed using the MEME program; a total of 10 conserved motifs were identified. The distribution of these 10 motifs in the SUT proteins is shown in Fig. 2. Motif 2 was the most conserved SUT domain and was identified in all of the SUT proteins except PeqSUT08 and DoSUT06.

In addition, motif 10 was observed in 17 SUT proteins but was absent in PeqSUT08, AsSUT05, DoSUT06, DoSUT08, and PeqSUT02. All three members in group A contained the same four motifs: motif 10, motif 2, motif 5 and motif 9. Moreover, except for DoSUT07, all group B members shared the same motif, motif 5; likewise, motif 4 was also common to all group B SUTs except for AsSUT04 (Fig. 2). Among the 12 SUTs in subgroup B2.1, three motifs were commonly present: motif 2, motif 3, and motif 5. There were eight sucrose transporters that had all 10 motifs, including five in *P. equestris* (PeqSUT03, PeqSUT04, PeqSUT05, PeqSUT06 and PeqSUT07) and two in *A. shenzhenica* (AsSUT02 and AsSUT06), whereas *D. officinale* had only one motif (DoSUT05). The sucrose transporters in each subgroup shared several unique motifs, indicating that the SUT proteins within the same subgroups may have certain functional similarities. In addition, the motif distribution of the SUTs suggested that these genes were largely conserved during evolution.

### Water-soluble sugar content in *D. officinale*

**Figure 3:**
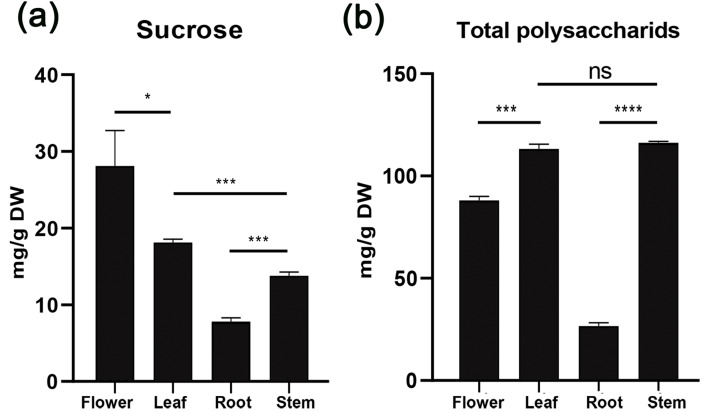
Histogram of water-soluble sugar content (mg/g) in different tissues of *D. officinale* including flower, stem, leaf and root. (A) Sucrose content. (B) Total polysaccharide content.

To understand sucrose partitioning and the functions of sucrose transporters in Orchidaceae species, we measured the water-soluble sugar content in different tissues of *D. officinale*, including leaves, stems, flowers and roots, using the GC-MS/MS method. The results showed that the content of water-soluble polysaccharides varied significantly among the different tissues (Table 2, Fig. 3). The amount of total water-soluble polysaccharides was highest in the stems of *D. officinale*, at approximately 116.17 mg/g, followed by the leaves, at approximately 113.23 mg/g. The flowers had approximately 88.08 mg/g, whereas the roots had a significantly lower level of water-soluble polysaccharides, at ∼26.66 mg/g (Fig. 3A). These results indicated that the water-soluble polysaccharides were mainly deposited in the stems of *D. officinale*. The sucrose content also varied greatly among the different tissues. Nonetheless, the sucrose content was highest in the flowers, at approximately 28.1 mg/g, followed by leaves (∼18.13 mg/g), which are the major source tissues for photosynthetically assimilated sucrose. The amount of sucrose in the stems was ∼13.77 mg/g, and that in roots was the lowest, at only ∼7.82 mg/g (Fig. 3B). Taken together, these results showed that although the total sugar content was highest in the stems, sucrose was mainly transported to the floral organs of *D. officinale*.

### Expression patterns of SUT genes in different tissues of *D. officinale*

**Figure 4:**
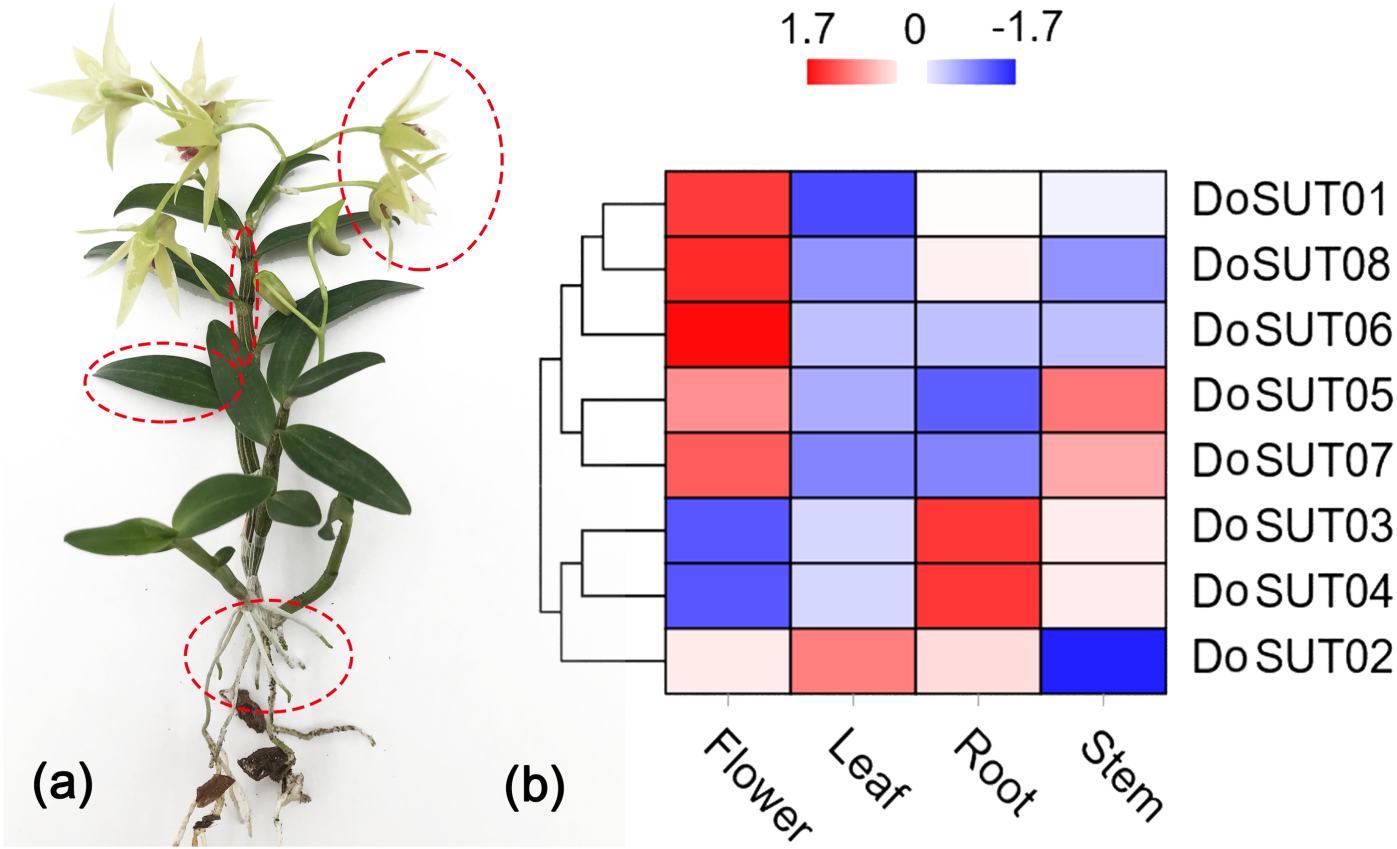
Expression of DoSUTs in different tissues. (A) An image of *D. officinale* used in this study. Red circles represent flowers, stems, leaves, and roots, respectively, which are used in transcriptome sequencing and qRT-PCR analysis. (B) Hierarchical clustering of gene expression profiles of *D. officnale SUTs* in different tissues including flowers, stems, leaves and roots. The FPKM values were visualized in the heat map.

To further understand the roles of *SUT* genes in orchids, the expression profiles of *DoSUT* genes in *D. officinale* were investigated. RNA sequencing (RNA-seq) was performed on different tissues, including the leaves, stems, flowers and roots, of *D. officinale*. The FPKM expression levels of the *DoSUT* genes in the four different tissues are provided in Table S2. Moreover, in Fig. 4, the expression levels of different *DoSUT* genes in the four *D. officinale* tissues are represented as different colors.

**Table 2:** Soluble sugar content (mg/g) in different tissues in *D. officinale*, including flower, root, and stem.

**Sample name**	**Sucrose content (mg/g)**	**Mean** ^a^	**Total sugar content (mg/g)**	**Mean** ^b^
Flowers	22.9	28.1	86.24303856	88.08374
	29.7		87.90367473	
	31.7		90.10451784	
Roots	8.37	7.816667	26.90351577	26.65843
	7.55		28.10009941	
	7.53		24.97167158	
Leaves	18.5	18.13333	110.6223197	113.2255
	17.7		114.3868356	
	18.2		114.6672478	
Stems	14.2	13.76667	115.7851793	116.1729
	13.2		117.0447804	
	13.9		115.6886072	

**Notes.**

a Mean value for sucrose content in different tissues.

b Mean values for total sugar content in different tissues.

In the present study, RNA-seq showed that most of the *DoSUTs* were expressed in the flowers, among which three genes, *DoSUT01*, *DoSUT08*, and *DoSUT06,* had significantly high expression levels. Phylogenetically, *DoSUT01*, *DoSUT08*, and *DoSUT06* were classified as members of subgroup A and of the monocot-specific expansion subgroup B2.1. Only one gene, *DoSUT02*, was significantly expressed in the leaves and was also expressed in the flowers and roots. We deduce that *DoSUT02* may play a role in phloem loading in *D. officinale*. Nonetheless, other sugar transporters, such as SWEETs and MSTs, are also likely involved in sucrose transport.

**Figure 5:**
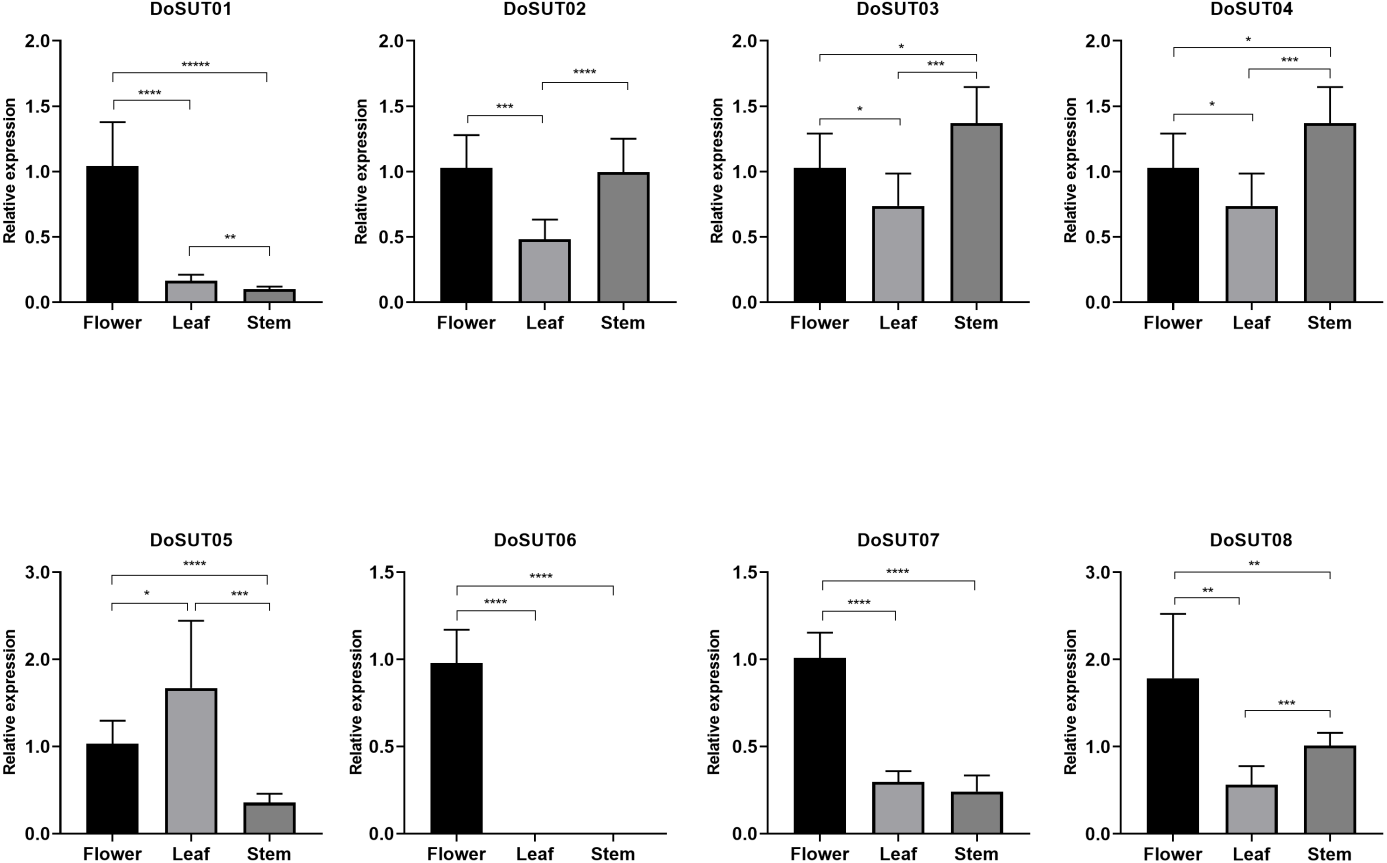
Expression levels of *DenSUT* genes in different tissues of *D. officinale* determined by qRT-PCR analysis. The results are shown as means ± SDs of three independent experiments. The presented gene expression levels are relative to the expression of the reference gene. An asterisk (*) indicates *P* value >0.05; two asterisks (**) indicate 0.01 < *P* < 0.05; *** indicates *P* < 0.01.

Three genes, *DoSUT03*, *DoSUT05* and *DoSUT07*, were expressed in the *Dendrobium* stems. Both *DoSUT05* and *DoSUT07* were moderately expressed in the stems and flowers, whereas *DoSUT03* was slightly expressed in the stems and significantly expressed in the roots. In addition, *DoSUT01* and *DoSUT08* were expressed at low levels in the roots. The expression of *DoSUTs* was also measured in the flowers, stems and leaves of *D. officinale* using qRT-PCR (Table S4, Fig. 5). The results were largely consistent with those from RNA-seq analysis. Specifically, *DoSUT01*, *DoSUT06*, *DoSUT07* and *DoSUT08* were significantly expressed in flowers. Nonetheless, *DoSUT02* was also expressed at significantly high levels in the stems. Three genes, *DoSUT03*, *DoSUT05* and *DoSUT07*, were expressed in *Dendrobium* stems. *DoSUT05* was expressed at significantly higher levels in the leaves than in the flowers, whereas *DoSUT03* was expressed at lower levels in the stems and at significantly higher levels in the roots.

## Discussion

Sucrose transporters are prevalent in plants and play fundamental roles in plant growth, development and stress tolerance (Braun & Barker, 2009; Yadav, Ayre & Bush, 2015; Lemoine et al., 2013). To date, a series of SUTs have been identified and characterized in plants; nonetheless, information on SUTs is still lacking for the Orchidaceae family, which is among the largest families of angiosperms. Orchids have vastly diverse morphotypes and lifestyles and exhibit extraordinary environmental adaptability; most are epiphytic, terrestrial or lithophytic, colonizing almost every habitat on Earth. Thus, they are excellent systems for elucidating the evolutionary history of angiosperms, especially floral organ evolution. In the present study, we performed a comprehensive analysis of the *SUT* gene families in three sequenced Orchidaceae species, which provided insights into the evolution, phylogenetics, and functions of *SUT* s in orchids and other plant species. In total, 22 *SUT* s were identified from *D. officinale*, *P. equestris*, and *A. shenzhenica*. Previous studies have indicated that plant sucrose transporters usually comprise 500–600 aa, with molecular weights of 55–60 kD (Reuscher et al., 2014; Getz et al., 1993). In this study, the molecular weights of the Orchidaceae SUTs varied between 51.22 and 106.90 kD, with lengths of ∼500 aa or ∼600 aa (Table 1).

To provide insights into the evolutionary patterns of *SUT* genes, we performed a phylogenetic analysis of 24 representative plant species. In recent studies, *SUTs* were classified into two subfamilies (Ancient Group 1 and Ancient Group 2) and three types (type I, type II and type III) (Reinders, Sivitz & Ward, 2012; Peng et al., 2014). The type I clade is dicot specific and corresponds to the SUT1 group (Kühn & Grof, 2010), and the type III clade contains SUTs of both monocots and dicots, which correspond to the SUT4 group (Lalonde & Frommer, 2012). Type II (A) is composed of SUTs from monocot and dicot species that were also reported in the SUT2 group by Lalonde & Frommer (2012), whereas monocot-specific Type IIB contains SUT3 and SUT5, as reported by Kühn & Grof (2010). We constructed a phylogenetic tree using the neighbor-joining method with 1,000 bootstraps. The *SUTs* from 24 representative plant species were classified into five subgroups: subgroups A, B1, B2.1, B2.2, and C (Fig. 1). Subgroups A and C contained members from mosses, lycophytes and both monocots and dicots. Subgroup B1 was dicot specific and corresponded to the SUT1 clade by Lalonde & Frommer (2012). Subgroup B2.2 was made up of members from both monocots and dicots, some of which could also be found in the SUT2 group, as reported by Lalonde & Frommer (2012). We found specific *SUT* gene expansion in some monocots in the monocot-specific subgroup B2.1, which also contained SUT3 and SUT5 (Kühn & Grof, 2010). We identified *SUTs* in primary terrestrial plants, including both lycophytes and mosses; however, none were identified in the green alga *Chlamydomonas reinhardtii* (Reinders, Sivitz & Ward, 2012). Moreover, *SUTs* were found to have expanded in monocot seed-bearing crop species such as maize (10 *SUTs*) and sorghum (8 *SUTs*) compared to *A. comosus* (3 *SUTs*). A total of 4-9 *SUTs* were identified in most dicot species. This conclusion is consistent with those of previous studies on *SUT* gene identification and evolution (Reinders, Sivitz & Ward, 2012; Lalonde & Frommer, 2012; Peng et al., 2014). The green algae *V. carteri* and *C. atmosphyticus* (Reinders, Sivitz & Ward, 2012) each contain one *SUT* homolog. According to previous studies, the SUT1 and SUT2 proteins mainly play roles in phloem loading and unloading, sucrose transport to sink cells, and sucrose exchange with microbes (Kühn & Grof, 2010; Doidy et al., 2012; Wittek et al., 2017; Barker et al., 2000; Milne, Grof & Patrick, 2018). SUT4 proteins are involved in various physiological processes, such as circadian rhythms and responses to dehydration and photosynthesis (Frost et al., 2012; Chincinska et al., 2013).

Photosynthetically produced sugars are not just carbon skeletons but also energy sources and signaling molecules that have major impacts on plant growth, development and physiology (Rolland, Baena-Gonzalez & Sheen, 2006; Julius et al., 2017). After being synthesized in mesophyll cells of leaves, sucrose needs to be loaded into phloem parenchyma cells or the apoplast of mesophyll cells and then transported through specialized networks [i.e., sieve element/companion cell complexes (SE/CCC)] and ultimately unloaded at distal sink organs (Kühn & Grof, 2010; Doidy et al., 2012; Julius et al., 2017). Unlike other monocot crop species such as maize, rice, and wheat, which use seeds as their main storage sink, the endosperm of most orchid seeds is significantly degenerated. As a result, Orchidaceae plants are highly dependent on symbiotic fungi to complete their life cycle, especially at the seed germination and seedling growth stages, due to nutrient deficiency (Yuan, Chen & Yang, 2009; Mccormick, Whijham & Canchani-Viruet, 2018; Rammitsu et al., 2019). We analyzed the water-soluble sugar content in *D. officinale* using the GC-MS/MS method, and the results showed that the content of total water-soluble polysaccharides was highest in the stems (∼116.17 mg/g), followed by the leaves (∼113.23 mg/g), flowers (∼88.08 mg/g), and roots (∼26.66 mg/g) (Fig. 3A). These results indicated that the stems were the major sink organs for sugar storage in *D. officinale.* Because *D. officinale* is an epiphytic plant species in its natural habitat that usually experiences drought stress (Zotz & Tyree, 1996; Wu, Raven & Hong, 2009), the high amount of sugar in the stems may help to maintain osmotic pressure to improve drought tolerance. However, the sucrose content was highest in the flowers (∼28.1 mg/g), followed by the leaves (∼18.13 mg/g), stems (∼13.77 mg/g), and roots (∼7.82 mg/g) (Fig. 3B). Previous studies have shown that developing pollen grains are strong sink tissues that require sucrose to provide energy for maturation, germination and growth (Stadler et al., 1999; Lemoine et al., 1999). Hence, although the total polysaccharides were mainly stored in the stems, sucrose was mainly transported to support the growth and physiology of the floral organs of *D. officinale*.

Sucrose transport systems play vital roles in carbon partitioning, plant development, inter-/intracellular communication and environmental adaptations. *SUT* genes not only are involved in sucrose transport but also play essential roles in pollen germination, fruit ripening, and ethylene biosynthesis in many species (Payyavula et al., 2011; Sivitz, Reinders & Ward, 2008; Srivastava et al., 2009a; Chincinska et al., 2013). *Arabidopsis AtSUC1* is expressed in seedlings where it is necessary for normal anthocyanin accumulation, whereas *AtSUC9* appears to be required for normal floral transition (Sivitz et al., 2007; Sivitz, Reinders & Ward, 2008). *AtSUC1* is also expressed in the parenchymatous cells of the style and anthers, which guides modulation of water availability around the region and ultimately results in pollen tubes moving toward the ovule and anther opening (Stadler et al., 1999). Recent studies have also described the roles of *NtSUT3* and *LeSUT2* in sucrose uptake during pollen development and pollen tube growth (Hackel et al., 2006; Lemoine et al., 1999). In this study, we conducted transcriptome sequencing on different tissues of *D. officinale* to determine the expression profiles and potential functions of *DoSUTs*. The results showed that most of the *DoSUTs* were expressed in the flowers; among them, *DoSUT01*, *DoSUT08*, and *DoSUT06* presented significantly increased expression levels. In agreement with the expression profiles, sucrose accumulation predominantly occurred in the flowers and reached approximately 28.1 mg/g. Taken together, these results indicated that these genes mainly function as part of the cellular machinery and development of floral organs.

In leaves, sucrose is mainly synthesized in mesophyll cell cytoplasm but may also be synthesized in organelles such as vacuoles and plastids (Schneider & Keller, 2009). Once released to the leaf apoplast, sucrose is actively loaded into SE-CCCs *via* a sucrose/H+ mechanism in apoplastic-loading species (Rennie & Turgeon, 2009). Analysis of transgenic and mutant plants indicates that dicot members of the SUT1 clade and monocot members of the SUT3 clade are essential for the apoplastic loading of SE-CCCs (Ishimaru et al., 2001; Gottwald et al., 2000; Slewinski, Meeley & Braun, 2009). In maize, *ZmSUT1* plays an important role in efficient phloem loading (Slewinski, Meeley & Braun, 2009). The inhibition of sucrose transporters results in starch accumulation in epidermal cells (Schulz et al., 1998). The sucrose transporter SUC2 is crucial for sucrose allocation; *Arabidopsis suc2* null mutants have compromised plant health (Srivastava et al., 2009b). After loading into the SE-CCC is occurs, energy-driving reloading is required throughout the whole process of long-distance sucrose transport from source to sink. In *D. officinale*, the sucrose content was ∼18.13 mg/g in the leaves, which ranked second among the four tissues. However, only one gene, *DoSUT02*, was significantly expressed in the leaves, which may have potential functions in phloem loading in *D. officinale*.

In well-studied grass stems, immature internodes are considered utilization sinks, whereas fully elongated mature internodes are storage sinks where sucrose accumulates (Hoffmann-Thoma et al., 1996; Rae, Perroux & Grof, 2005; Bihmidine et al., 2015). Plasma membrane-localized sucrose transporters are promising candidates for sucrose uptake in stems. For example, all of the *SbSUTs* in sorghum are active in sucrose uptake, although the expression sites of different *SUTs* in internodes may vary (Braun & Barker, 2009; Bihmidine et al., 2015; Martin et al., 2016). *SbSUTs* are localized to sieve elements in both developing and mature sorghum stems (Milne et al., 2017), which is consistent with the localization of wheat *TaSUT1* and rice *OsSUT1* proteins in SE-CCCs in mature stems (Aoki et al., 2004; Scofield et al., 2007). In the present study, four genes (*DoSUT03*, *DoSUT04*, *DoSUT05* and *DoSUT07*) may function in sucrose transport in *Dendrobium* stems. However, the specific functions of *SUT* genes in *D. officinale* and other Orchidaceae species remain unknown.

## Conclusions

In conclusion, we performed a comprehensive study of the phylogenetic relationships of the SUTs in 24 plant species and a genome-wide characterization of the *SUT* genes in three Orchidaceae species. The *SUTs* were classified into three groups and five subgroups. We identified a total of 22 *SUT* genes in three orchid species: eight *DoSUTs*, eight *PeqSUTs*, and six *AsSUTs*. The functions of the *SUTs* in *Dendrobium* were analyzed. The results showed that most of the *DoSUTs* were highly expressed in the flowers. Although the total content of water-soluble sugars was highest in the stems, the sucrose content was highest in the flowers. We propose that stems are used as major sinks for sugar storage in *D. officinale* and that *DoSUTs* mainly function in floral organs. Our findings provide important insights into the evolutionary patterns of plants and advance our knowledge of sucrose partitioning and of the potential functions of *SUT* genes in Orchidaceae species.

## Supplemental Information

10.7717/peerj.11961/supp-1 Supplemental Information 1 Protein sequence of the 22 SUTs from *A. shenzhennica*, *D. officinale*, and *P. equestris*

10.7717/peerj.11961/supp-2 Supplemental Information 2 FPKM expression of *DenSUT* genes in four different tissues of *D. officinale*

10.7717/peerj.11961/supp-3 Supplemental Information 3 Primer sequences used in qRT-PCR analysis

10.7717/peerj.11961/supp-4 Supplemental Information 4 Relative expression for eight *DenSUT* s in flowers, roots, and stems in *D. officinale* using qRT-PCR

## Abbreviation List

SUTs/SUCs: Sucrose transporters
TM: Transmembrane
MEME: Multiple Em for Motif Elicitation
SE: Sieve element
CCC: Companion cell complex
SWEET: Sugars Will Eventually be Exported Transporter
MSTs: Monosaccharide transporters
RNA-seq: RNA sequencing
qRT-PCR: Quantitative real-time polymerase chain reaction

## Additional Information and Declarations

### Data Availability

The following information was supplied regarding data availability: The raw data of the RNA-seq experiment is available in Sequence Read Archive (NCBI): PRJNA680456.
